# Physiological and Proteomic Responses to Chronic Stress and the Effects of *Chrysanthemum zawadskii* var. *latilobum* (Maxim.) Kitam. Extract in Beagle Dogs

**DOI:** 10.3390/ani16172679

**Published:** 2026-08-26

**Authors:** Ye Jin Yang, Min Jung Kim, Hee Ho Kim, Ji Woong Heo, Ju Lan Chun, Eui-Cheol Shin, Kyeong Soo Kim, Hyun-Wook Kim, Eun Ju Jeong, Dongbin Lee, Kwang Il Park

**Affiliations:** 1 College of Veterinary Medicine, Gyeongsang National University, Jinju 52828, Republic of Korea; yang93810@gnu.ac.kr (Y.J.Y.); minjung0102@gnu.ac.kr (M.J.K.); hiho2323@gnu.ac.kr (H.H.K.); hujiw7806@gnu.ac.kr (J.W.H.); 2 Animal Welfare Division, National Institute of Animal Science, 1500 Kongjwipatjwi-ro, Iseomyeon, Wanju-gun 55365, Republic of Korea; julanchun@korea.kr; 3 Food Science and Technology, Gyeongsang National University, Jinju 52828, Republic of Korea; eshin@gnu.ac.kr; 4 Department of Pharmaceutical Engineering, Gyeongsang National University, 501, Jinju-daero, Jinju 52725, Republic of Korea; soyoyu79@gnu.ac.kr; 5 Division of Animal Bioscience & Integrated Biotechnology, Gyeongsang National University, Jinju 52828, Republic of Korea; hwkim@gnu.ac.kr; 6 Agri-Food Bio Convergence Institute, Gyeongsang National University, Jinju 52725, Republic of Korea; jeong.ej@gnu.ac.kr; 7 Department of Veterinary Surgery, Gyeongsang National University, Jinju 52725, Republic of Korea; dlee@gnu.ac.kr

**Keywords:** chronic stress, beagle dog, serum proteomics, stress biomarkers, *Chrysanthemum zawadskii* var. *latilobum* (Maxim.) Kitam

## Abstract

Chronic stress activates the hypothalamic–pituitary–adrenal (HPA) axis and induces systemic physiological changes. This study aimed to identify stress-associated serum biomarker candidates in chronically stressed beagle dogs and to evaluate the anti-stress effects of *Chrysanthemum zawadskii* var. *latilobum* (Maxim.) Kitam. extract (CZE). Beagle dogs were subjected to movement restriction-induced chronic stress for 42 days, with or without repeated oral administration of CZE. Serum cortisol and corticosterone concentrations were measured using ELISA, and hematological and serum biochemical parameters were analyzed. Serum samples collected at day 0 and day 42 of the 84-day chronic stress model were subjected to LC-MS/MS-based proteomic analysis. Chronic stress increased serum cortisol and corticosterone concentrations and induced alterations in hematological and serum biochemical parameters, whereas CZE administration tended to attenuate or stabilize these stress-associated changes. Proteomic analysis identified 380 serum proteins, of which 348 quantified proteins were subjected to differential expression analysis. The differentially expressed proteins were primarily associated with immune response, inflammatory response, extracellular matrix organization, angiogenesis, neurogenesis, and secretion. TIMP1, TREM2, RAB8A, KRT9, PPBP, THBS1, and HSPB1 were identified as candidate stress-associated serum biomarkers. These findings indicate that chronic stress induces endocrine, hematological, biochemical, and serum proteomic alterations in beagle dogs. Changes in proteins related to immune–inflammatory regulation and extracellular matrix organization suggest that prolonged stress may affect systemic immune responses and tissue remodeling. CZE may contribute to the maintenance of physiological homeostasis under chronic stress conditions and may have potential as a functional anti-stress material, while the identified serum proteins may provide a basis for the development of biomarkers for chronic stress.

## Simple Summary

This study investigated the physiological and molecular changes induced by chronic stress in beagle dogs and evaluated the potential anti-stress effects of *Chrysanthemum zawadskii* var. *latilobum* (Maxim.) Kitam. extract (CZE). Chronic stress increased circulating cortisol and corticosterone levels and altered hematological, serum biochemical, and proteomic profiles. Serum proteomic analysis identified TIMP1, TREM2, RAB8A, KRT9, PPBP, THBS1, and HSPB1 as potential stress-associated biomarker candidates. Repeated oral administration of CZE tended to attenuate or stabilize several stress-related physiological changes. These findings suggest that CZE may have potential as a functional anti-stress material and that the identified serum proteins may contribute to the development of biomarkers for chronic stress.

## 1. Introduction

Stress is a complex physiological and psychological response that occurs when an organism is exposed to internal or external stimuli and attempts to maintain homeostasis [1,2,3]. Acute stress activates the sympathetic nervous system and induces a “fight-or-flight” response, characterized by increased muscle tension, elevated blood pressure, and increased heart rate [3]. This response is primarily mediated by catecholamines, including epinephrine and norepinephrine [4]. Although acute stress serves as an adaptive defense mechanism against external stimuli, prolonged or repeated stress can disrupt the regulation of the central nervous, autonomic, and endocrine systems, ultimately leading to impaired physiological homeostasis [5,6].

Chronic stress persistently activates the hypothalamic–pituitary–adrenal axis (HPA axis), resulting in increased secretion of glucocorticoids, particularly cortisol, from the adrenal cortex [7,8,9]. Elevated glucocorticoids promote energy mobilization by modulating protein and lipid metabolism [10]; however, sustained glucocorticoid elevation may impair immune homeostasis, disrupt inflammatory regulation, and induce metabolic disturbances [11]. In this context, chronic stress is recognized as an important factor contributing to the onset and progression of various diseases by promoting immunosuppression, increasing susceptibility to infection, and, in some cases, exacerbating immune activation and inflammation [12].

Sustained exposure to stress is associated with a wide range of physiological and pathological outcomes, ranging from relatively mild symptoms such as headache and irritable bowel syndrome to more severe conditions, including cognitive impairment, chronic fatigue, depression, metabolic disorders, and immune dysfunction [13,14,15]. Over time, these stress-induced alterations may increase the risk of chronic diseases such as gastric ulcers, diabetes, hypertension, cardiovascular diseases, liver diseases, and cancer, or aggravate pre-existing disease conditions [16,17,18]. Therefore, the identification of objective physiological indicators and stress-specific biomarkers is essential for the early assessment, prevention, and management of stress-related disorders [19].

In animal-based stress research, movement restriction-based models have been used to induce reproducible biochemical and physiological stress responses by limiting animal movement and behavior [7,20]. These models are useful for evaluating stress-associated hormonal, hematological, and metabolic alterations [7]. However, serum protein changes induced by long-term stress and their associations with stress-related physiological indicators remain insufficiently characterized. Thus, serum proteomic analysis may provide a valuable approach for understanding the molecular mechanisms underlying chronic stress and for identifying novel biomarker candidates [21,22,23].

*Chrysanthemum zawadskii* var. *latilobum* (Maxim.) Kitam. (CZ) is a perennial plant belonging to the family Asteraceae and has traditionally been used as a medicinal plant with anti-inflammatory, analgesic, antioxidant, and immunomodulatory properties [24]. This plant contains flavonoids, phenolic compounds, and other bioactive constituents that may contribute to the regulation of oxidative stress and inflammatory responses [25,26]. Considering that chronic stress is associated with oxidative damage, inflammatory dysregulation, and immune imbalance, natural products with antioxidant and anti-inflammatory activities may represent potential candidates for mitigating stress-associated physiological alterations [13,27]. However, the effects of CZE on hematological alterations and serum stress-related markers under prolonged stress conditions remain unclear.

In this study, we established a movement restriction-based chronic stress model in beagle dogs and monitored serum glucocorticoid levels during the stress induction period to determine the time point at which the stress response was most evident. Based on this assessment, serum samples collected on day 42 were subjected to proteomic analysis to identify proteins altered under chronic stress conditions and to discover candidate stress-associated biomarkers. In addition, CZE was orally administered to beagle dogs to evaluate its anti-stress efficacy under prolonged stress conditions. Hematological, serum biochemical, and glucocorticoid parameters were comprehensively assessed to determine whether CZE could attenuate chronic stress-induced physiological and biochemical alterations, thereby evaluating its potential as a functional anti-stress material.

## 2. Materials and Methods

### 2.1. Preparation of CZE

Dried CZ was purchased from Dongeuihanjae at Seoul Yangnyeongsi Market (Dongdaemun-gu, Seoul, Republic of Korea). The dried CZ was authenticated by Professor Ki Hwan Bae, a medical botanist at the College of Pharmacy, Chungnam National University, Republic of Korea, and the whole aerial parts were used for extraction. The dried CZ was finely pulverized into powder and extracted with 20 volumes of 40% ethanol (Sigma-Aldrich, St. Louis, MO, USA) at 70 °C for 4 h in a water bath. The extract was filtered through a 1 μm filter and subsequently concentrated under reduced pressure at 60 ± 5 °C to obtain a concentrate of 25 °Brix. The concentrate was sterilized at 90 ± 5 °C for 30 min, frozen at −40 °C, and then lyophilized for 72 h to obtain a dried powder. The lyophilized powder was further ground and passed through a 40-mesh sieve to produce a homogeneous CZ 40% ethanol extract powder. The final extraction yield was 14.7% based on the dried raw material. The prepared CZ 40% ethanol extract was named CZE and stored at −20 °C until use.

### 2.2. Preparation and Evaluation of CZE Tablets

CZE tablets were prepared by dry granulation without the addition of a binder solution or granulating solvent. The formulation composition of the CZE tablets is presented in Supplementary Table S1. Briefly, the excipients, excluding CZE and magnesium stearate, were accurately weighed and manually mixed in a polyethylene mixing bag until a homogeneous powder blend was obtained. The mixture was then passed through a 30-mesh sieve with an aperture size of 600 μm. CZE was added to the sieved excipient mixture and manually blended until uniformly mixed. Magnesium stearate was then added as a lubricant and mixed for less than 2 min to avoid over-lubrication. The final blend was compressed into tablets using a rotary tablet press machine (ZP10; M.D Korea Co., Hwaseongsi, Republic of Korea) equipped with a round punch with a diameter of 10.8 mm. The compression speed was set at 25 rpm, and the tablet hardness was adjusted to 10–12 kp. The prepared tablets were evaluated for their physical properties, including appearance, weight variation, hardness, friability, and time disintegration. Tablet friability was measured using a friability tester (CS-4; NANBEI, Zhengzhou, China). The tablets were weighed before and after the friability test, and friability was calculated as the percentage of weight loss. Disintegration time was determined using a disintegration tester (KDIT-200; Kukje Engineering, Goyang, Republic of Korea), and the time required for complete disintegration was recorded.

### 2.3. Animals and Ethical Approval

All beagle dogs (*Canis lupus familiaris*) weighing 9–14 kg were used in the present study and were housed in groups at the animal facility of Gyeongsang National University under controlled environmental conditions. The dogs had already been maintained at the same facility before study initiation; therefore, no additional acclimatization period was required. The room temperature was maintained at 23 ± 2 °C, with a relative humidity of 40–60% and a 12 h light/12 h dark cycle, with lights on from 6:00 a.m. to 6:00 p.m. The dogs were fed once daily and had ad libitum access to drinking water. Detailed information on all beagle dogs used in this study is provided in Tables S2 and S3. All animal experimental procedures were reviewed and approved by the Institutional Animal Care and Use Committee (IACUC) of Gyeongsang National University. The chronic stress model establishment study and the CZE efficacy study were approved under Approval No. GNU-230522-D0101 and GNU-250604-D0123, respectively. All experiments were conducted in accordance with institutional guidelines for the care and use of laboratory animals.

### 2.4. Establishment of an 84-Day Chronic Stress Model in Beagle Dogs

**Figure 1:**
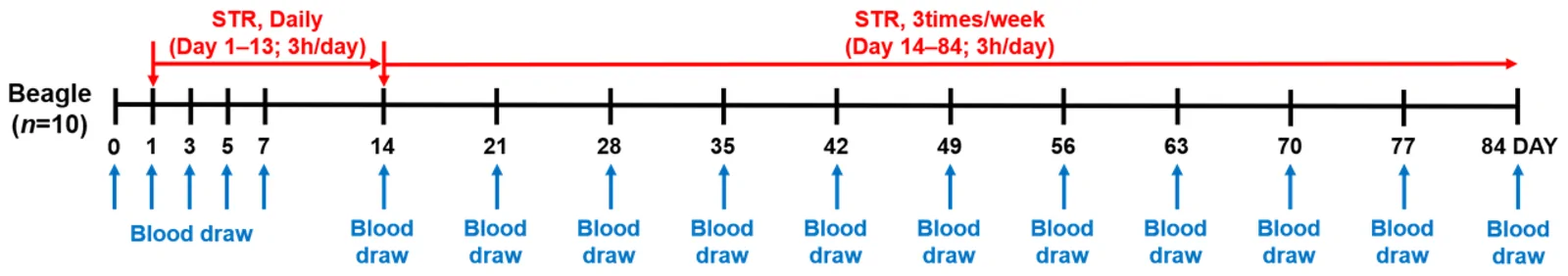
Experimental schedule for establishment of the 84day chronic stress model in beagle dogs. Day 0 was defined as the baseline before stress induction. Stress was induced for 3 h per day from day 1 to day 13 and thereafter three times per week (Monday, Wednesday, and Friday) from day 14 to day 84. Blood samples were collected on days 0, 1, 3, and 5 during the first week and then once weekly on Monday from day 7 to day 84, as indicated by the blue arrows. STR, stress induction.

In this study, 10 healthy beagle dogs were used to establish an 84-day chronic stress model (Table S2). The first blood sampling time point before stress induction was defined as day 0 (baseline), and stress induction and blood collection were performed over a total period of 12 weeks. Stress was induced for 3 h per day, from 10:00 to 13:00. From day 1 to day 13, stress was applied daily, whereas from day 14 to day 84, it was applied three times per week on Monday, Wednesday, and Friday. Blood samples were collected at baseline (day 0) and on days 1, 3, and 5 during the first week. From day 7 to day 84, blood samples were collected once weekly on Monday (Figure 1). On Mondays during the stress-induction period, blood collection was performed immediately after completion of the 3 h stress procedure. All blood sampling procedures were conducted by an experienced professional with a veterinary license. Blood was collected from the jugular vein of each beagle dog using a syringe and transferred into appropriate tubes according to the purpose of each analysis, including ethylenediaminetetraacetic acid (EDTA) tubes, heparin tubes, or 1 mL tubes. To minimize animal burden caused by repeated blood collection, the sampling volume was determined according to the criteria described in the Good Practice Guide for Blood Sampling [28]. The appropriate blood volume was calculated based on the body weight of each beagle dog and the overall experimental duration, and excessive blood collection was carefully avoided throughout the study period.

### 2.5. Evaluation of CZE Efficacy in Chronically Stressed Beagle Dogs

**Figure 2:**
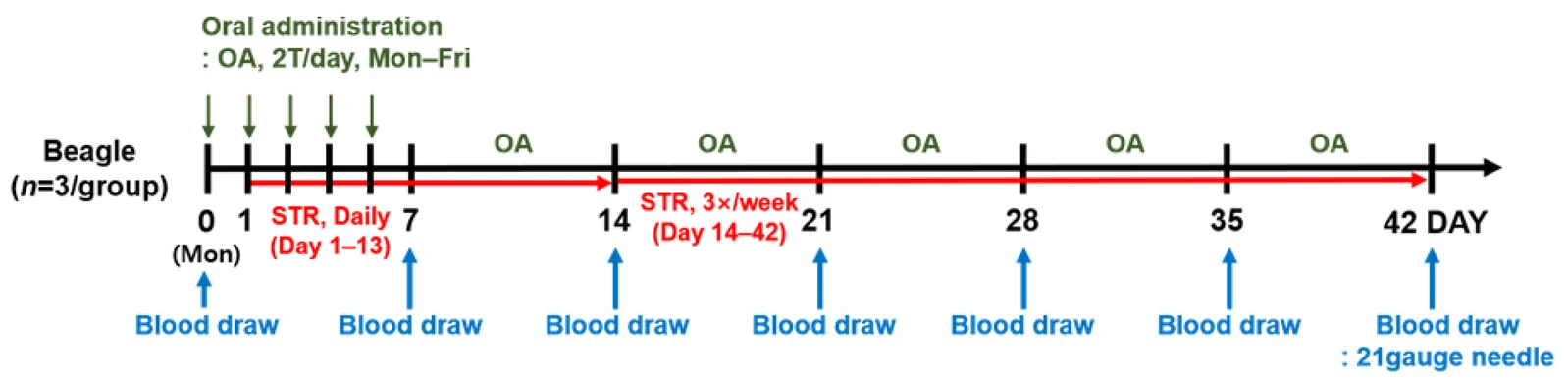
Experimental design of the 42-day CZE treatment experiment in beagle dogs. Beagle dogs were subjected to chronic stress through movement restriction in a cage (40 × 45 × 65 cm) for 3 h/day, daily from day 1 to day 13 and three times per week (Monday, Wednesday, and Friday) from day 14 to day 42. CZE was orally administered at a dose of two tablets per day, five days per week (Monday to Friday), from day 1 to day 42. Blood samples were collected at the indicated time points, as shown by the blue arrows. Red arrows indicate stress induction, and green arrows indicate oral administration of CZE. STR, stress induction; OA, oral administration; CZE, *Chrysanthemum zawadskii* var. *latilobum* (Maxim.) Kitam extract.

To evaluate the stress-suppressive effects of CZE, 6 beagle dogs were used in a 42-day CZE treatment experiment and divided into the stress-induced group (STR, *n* = 3) and the CZE-treated stress group (CZE + STR, *n* = 3). Allocation of dogs to the STR and CZE + STR groups was performed using a completely randomized design. The beagle dog stress model was established for 42 days. From day 1 to day 42, CZE in tablet form was orally administered to the CZE + STR group before stress induction at a dose of two tablets per administration (200 mg CZE contained in each 460 mg tablet), five times per week from Monday to Friday. During the initial 2 weeks, from day 1 to day 13, stress was induced daily for 3 h from 10:00 to 13:00. Thereafter, from day 14 to day 42, stress was induced three times per week on Monday, Wednesday, and Friday during the same 3 h period. Blood samples were collected at baseline (day 0) before stress induction and thereafter once weekly on Monday from day 7 to day 42. On scheduled blood-sampling days, blood was collected immediately after completion of the stress-induction procedure. Blood collection was performed by an experienced veterinarian. Blood was collected from the jugular vein of each beagle dog using a syringe and transferred into appropriate tubes according to the purpose of each analysis, including EDTA tubes, heparin tubes, or 1 mL tubes. To minimize the animal burden associated with repeated blood sampling, blood collection was performed in accordance with the Good Practice Guide for Blood Sampling [28]. The experimental design is shown in Figure 2, and the characteristics of the beagle dogs used in the CZE efficacy study are summarized in Supplementary Table S3.

### 2.6. Complete Blood Count Analysis

Complete blood count (CBC) analysis was performed to evaluate the overall health status of beagle dogs following stress induction and drug administration. Fresh blood samples were collected in EDTA tubes, and cellular blood components, including red blood cells, white blood cells, and platelets, were analyzed using an automatic blood cell counter (IDEXX ProCyte Dx Hematology Analyzer; IDEXX Laboratories, Inc., Westbrook, ME, USA). The analyzed parameters are listed in Table S4.

### 2.7. Serum Chemistry Analysis

Serum chemistry analysis was performed to assess major organ function and metabolic status following stress induction and drug administration. Blood samples were collected in serum-separating tubes (SST), and biochemical parameters were measured using an automatic chemistry analyzer (Catalyst One Chemistry Analyzer; IDEXX Laboratories, Inc., Westbrook, ME, USA). The analyzed parameters are listed in Table S4.

### 2.8. Enzyme-Linked Immunosorbent Assay Analysis

Blood samples collected from beagle dogs were incubated at room temperature for 2 h and then centrifuged at 3000 rpm for 15 min. The supernatant was collected to obtain serum. Serum cortisol and corticosterone concentrations were measured using canine-specific ELISA kits for cortisol (K003-H5, Arbor Assays, Ann Arbor, MI, USA) and corticosterone (K014-H5, Arbor Assays, USA), respectively. All serum samples were diluted at a final ratio of 1:100 prior to analysis. Briefly, serum samples were first mixed with dissociation reagent (DR) at a 1:1 ratio and incubated at room temperature for approximately 10 min. The mixture was then further diluted 50-fold using 1× assay buffer (Arbor Assays, Ann Arbor, MI, USA) to achieve a final 1:100 dilution. Three serum samples per group were analyzed, and the results were calculated and presented as the mean value.

### 2.9. Protein Preparation and Digestion

Serum samples collected at baseline (day 0) and on day 42 of the 84-day chronic stress-model establishment experiment were subjected to proteomic analysis. Serum protein concentrations were quantified using the Pierce BCA Protein Assay Kit (Thermo Fisher Scientific, Waltham, MA, USA). Protein digestion was performed using the filter-aided sample preparation (FASP) method with Microcon 30 K centrifugal filter devices (Millipore, Billerica, MA, USA). For each sample, 100 μg of protein was reduced with 5 mM tris(2-carboxyethyl) phosphine (TCEP) at 37 °C for 30 min and subsequently alkylated with 50 mM iodoacetic acid (IAA) at 25 °C for 1 h in the dark. After sequential washing with lysis buffer and 50 mM ammonium bicarbonate (ABC; Sigma-Aldrich, St. Louis, MO, USA), the proteins were digested with trypsin at an enzyme-to-protein ratio of 1:50 (*w*/*w*) at 37 °C for 18 h. The resulting peptide mixtures were collected, and trypsin activity was terminated by acidification with 15 μL of formic acid (Honeywell, Charlotte, NC, USA). The digested peptides were desalted using C18 spin columns (Harvard Apparatus, Holliston, MA, USA), eluted with 80% acetonitrile (Sigma-Aldrich, St Louis, MO, USA) in 0.1% formic acid (Honeywell, Charlotte, NC, USA), and dried using a centrifugal vacuum concentrator (Thermo Fisher Scientific, Waltham, MA, USA).

### 2.10. LC-MS/MS Analysis

The prepared peptide samples were analyzed using an Orbitrap Exploris 480 mass spectrometer coupled to an UltiMate 3000 RSLC Nano LC system (Thermo Fisher Scientific, Bremen, Germany). Peptides were loaded onto a 2 cm × 100 μm ID trap column packed with 3 μm C18 resin and separated using a 15 cm × 75 μm ID analytical column packed with 3 μm C18 resin. The mobile phases consisted of 0.1% formic acid in 5% DMSO (solvent A) and 0.1% formic acid in 5% DMSO containing 80% acetonitrile (solvent B). The flow rate was maintained at 250 nL/min. The gradient was programmed as follows: 5–40% solvent B for 150 min, 40–95% solvent B for 2 min, 95% solvent B for 23 min, 95–5% solvent B for 10 min, and 5% solvent B for 15 min. MS data were acquired using a data-dependent acquisition mode, and the top 30 precursor ions were selected and isolated for fragmentation. The monoisotopic precursor selection (MIPS) peptide algorithm was applied with relaxed restrictions when an insufficient number of precursors met the selection criteria. MS1 and MS2 spectra were acquired at resolutions of 60,000 and 15,000, respectively, at *m*/*z* 400, and the MS1 scan range was set to 350–1800 *m*/*z*. Precursor ions were fragmented using a normalized collision energy (NCE) of 27%, and dynamic exclusion was set to 40 s. Detailed LC-MS/MS conditions are provided in Supplementary Table S5.

### 2.11. Proteome Data Analysis

LC-MS/MS raw data were analyzed using Proteome Discoverer software (version 2.5). MS/MS spectra were searched against the canine protein database obtained from UniProt using SEQUEST HT as the database search algorithm, and peptide-spectrum matches (PSMs) were subjected to validation. The precursor ion mass tolerance was set to 10 ppm, and the fragment ion mass tolerance was set to 0.02 Da. Trypsin was specified as the proteolytic enzyme, allowing a maximum of two missed cleavages. Carbamidomethylation of cysteine (+57.012 Da) was set as a fixed modification, whereas oxidation of methionine (+15.995 Da), acetylation of the protein N-terminus (+42.011 Da), and carbamylation of the protein N-terminus (+43.0006 Da) were set as variable modifications. Peptide-spectrum matches were filtered using a false discovery rate (FDR) of <1%, and peptides with a minimum length of six amino acids were retained for further analysis. Detailed database search parameters are provided in Supplementary Table S6.

### 2.12. Differential Protein Abundance Analysis

Differential protein abundance between day 0 and day 42 of the 84-day chronic stress model was analyzed using ExDEGA version 5.1.1 (E-biogen Inc., Seoul, Republic of Korea). Proteins with an absolute log2 fold change (|log_2_FC|) > 1 and a *p* value < 0.05 were considered differentially abundant proteins. A volcano plot was generated to visualize changes in protein abundance at day 42 compared with day 0, and hierarchical clustering analysis was performed to visualize differences in serum protein abundance patterns between the two time points. Functional annotation of the differentially abundant proteins was further performed by classifying the proteins according to their associated biological processes. Gene category information used for functional classification was obtained from QuickGO (https://www.ebi.ac.uk/QuickGO/ accessed on 23 March 2025). The proteins were categorized into functional groups, including angiogenesis, apoptotic process, cell cycle, cell differentiation, cell migration, extracellular matrix, immune response, inflammatory response, neurogenesis, secretion, and cellular response to stress. The proportions of upregulated and downregulated proteins within each functional category were analyzed and visualized using ExDEGA. In addition, a circular heatmap of the selected candidate proteins was generated using the SRplot platform (https://bioinformatics.com.cn/srplot accessed on 10 April 2025).

### 2.13. Protein–Protein Interaction (PPI) Network Analysis

Protein–protein interaction (PPI) networks among the differentially abundant proteins were constructed using STRING v12.0 (https://string-db.org/ accessed on 10 April 2025), with *Canis lupus familiaris* selected as the reference organism. A minimum interaction confidence score of 0.9 (highest confidence) was applied. Functional modules within the PPI network were identified using the Markov Cluster Algorithm (MCL) with an inflation parameter of 2.0 to characterize major protein interaction clusters.

### 2.14. Statistical Analysis

All statistical analyses were performed using GraphPad Prism version 8.0 (GraphPad Software, San Diego, CA, USA). Data are presented as the mean ± standard error of the mean (SEM). Time-course data were analyzed using one-way and two-way repeated-measures analysis of variance (ANOVA) to evaluate the effects of treatment, time, and their interaction. Dunnett’s multiple comparisons test was used to compare each subsequent time point with baseline (day 0) within each group, whereas Sidak’s multiple comparisons test was used to compare the STR and CZE + STR groups at each time point. A *p* value < 0.05 was considered statistically significant. Adjusted *p* values from the multiple comparisons tests are reported where appropriate. An a priori power analysis was performed using G*Power software (version 3.1.9.7; Heinrich-Heine-Universität Düsseldorf, Düsseldorf, Germany) to estimate the minimum sample size required for the study. Based on a significance level of α = 0.05 and a target statistical power of 70%, the minimum required sample size was estimated to be three dogs per group. In addition, a post hoc power analysis based on the observed data indicated a statistical power exceeding 90%.

## 3. Results

### 3.1. Change in Cortisol and Corticosterone Levels

**Figure 3:**
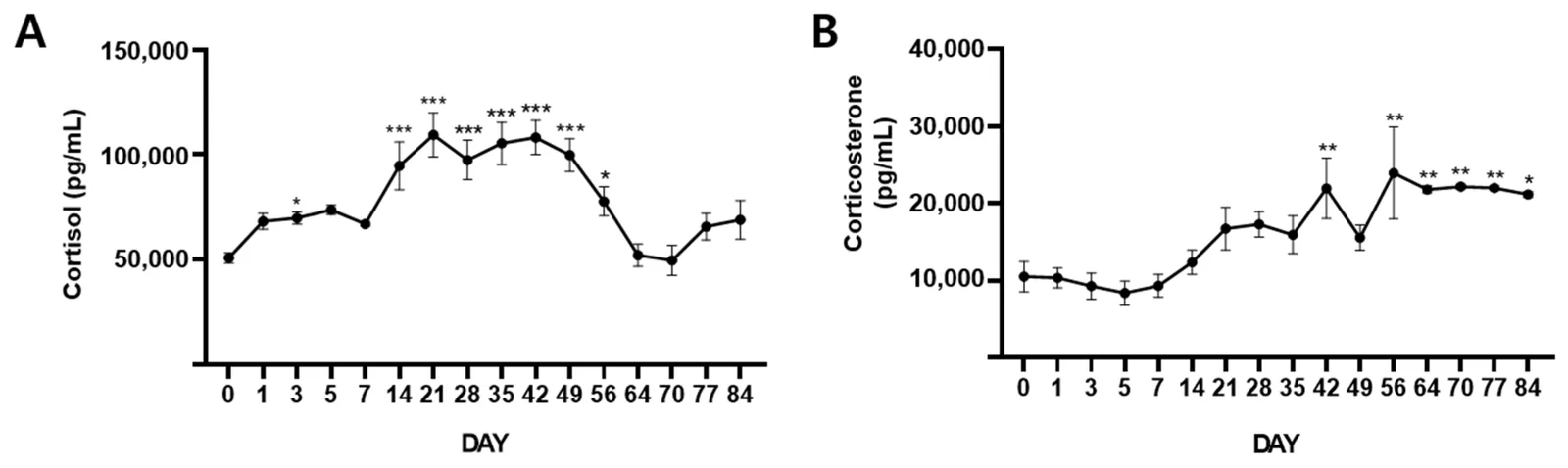
Increasing cortisol and corticosterone levels according to the experimental period in stress-induced beagle dogs. (**A**) The level of cortisol gradually increased from day 0 (baseline) to day 42 (*n* = 10). (**B**) The level of corticosterone tended to increase from day 0 (baseline) to day 42 (*n* = 10). Data were analyzed by one-way ANOVA followed by Dunnett’s multiple comparisons test against STR day 0. * *p* < 0.05, ** *p* < 0.01, and *** *p* < 0.001 vs. STR day 0.

To evaluate changes in circulating hormone levels following chronic stress induction in the 84-day chronic stress model, and drug administration, serum cortisol and corticosterone concentrations were measured. Serum cortisol levels tended to increase compared with day 0 as the experimental period progressed, showing a continuous elevation up to day 42 (Figure 3A; Day 14; *p* < 0.001, Day 21; *p* < 0.001, Day 28; *p* < 0.001, Day 35; *p* < 0.001, Day 42; *p* < 0.001). Although cortisol levels transiently decreased thereafter, an increasing trend was observed again after day 70. Serum corticosterone levels also showed a time-dependent increase and continued to rise to day 84 (Figure 3B, Day 70; *p* = 0.010, Day 77; *p* = 0.008, Day 84; *p* = 0.017). Both cortisol (Figure 3A; Day 42, *p* < 0.001) and corticosterone (Figure 3B; Day 42, *p* = 0.004) exhibited a sustained increasing trend during the early phase of the experimental period, particularly up to day 42. Based on these results, day 42 was selected as a representative time point at which the stress response was stably induced, and subsequent analyses were performed using this time point from the 84-day chronic stress model.

### 3.2. Serum Proteomic Profiling Under Chronic Stress Conditions

**Figure 4:**
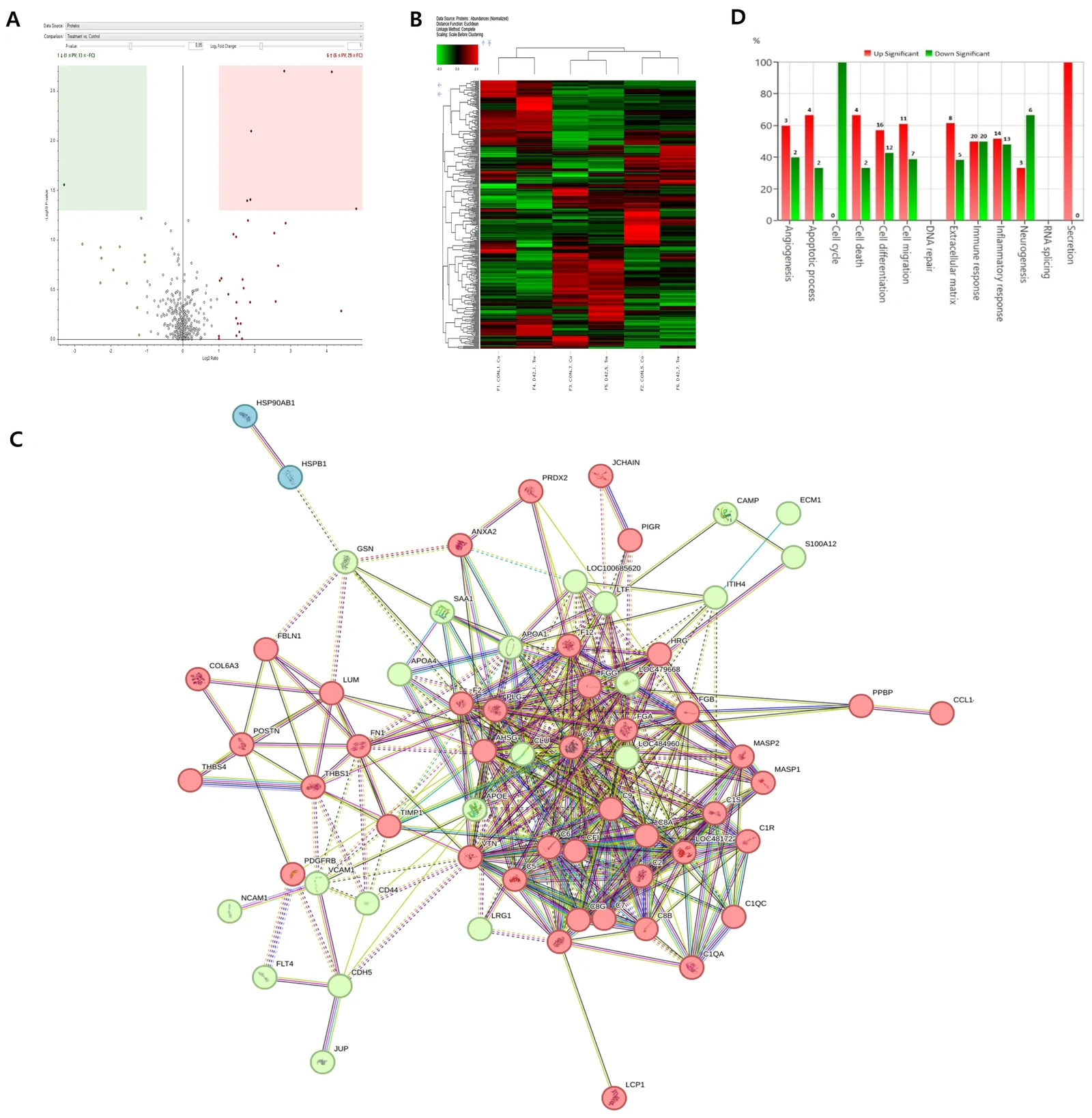

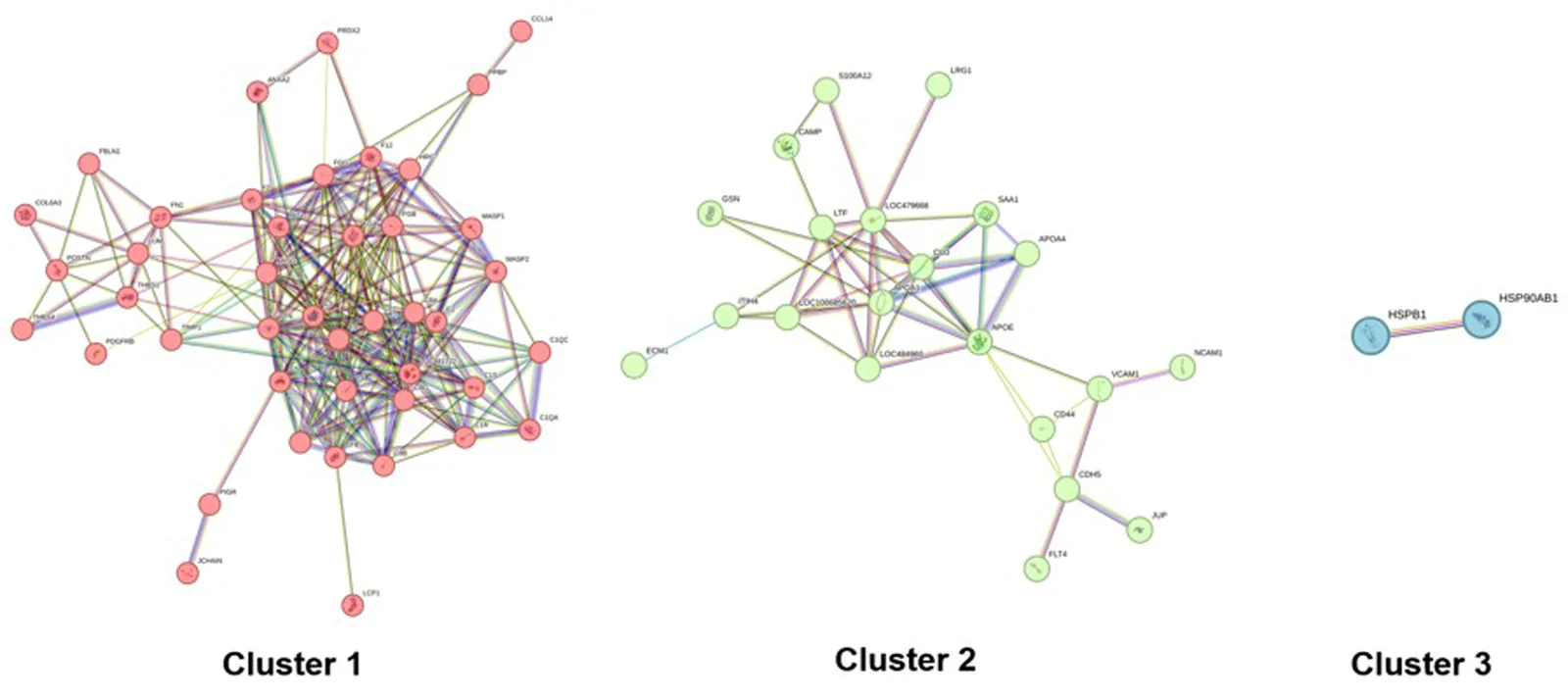
Serum proteomic profiling under chronic stress conditions. Serum samples collected at day 0 and day 42 of the 84-day chronic stress model were subjected to LC-MS/MS-based proteomic analysis. (**A**) Volcano plot showing differentially abundant proteins at day 42 compared with day 0. Proteins with ∣log_2_FC∣ > 1 and *p* < 0.05 were considered significantly altered. (**B**) Hierarchical clustering heatmap showing differences in serum protein abundance patterns between day 0 and day 42. (**C**) Protein–protein interaction network constructed using the STRING database. The upper panel shows the overall interaction network, and the lower panel shows three major interaction clusters identified within the network. (**D**) Functional classification of differentially abundant proteins according to biological process categories. Red and green bars indicate the proportions of upregulated and downregulated proteins, respectively.

Based on the ELISA results, LC-MS/MS-based proteomic analysis was performed using serum samples collected at day 0 and day 42 of the 84-day chronic stress model. A total of 380 proteins were identified across the serum samples analyzed at day 0 and day 42, and 348 quantified proteins were further analyzed to determine stress-induced changes in protein abundance at day 42 compared with day 0. Volcano plot analysis revealed multiple proteins that were significantly up- or down-regulated at day 42 compared with day 0 (Figure 4A). In addition, hierarchical clustering heatmap analysis demonstrated distinct differences in protein abundance patterns between day 0 and day 42 (Figure 4B), indicating that chronic stress altered the serum proteomic profile in beagle dogs. To investigate the functional interactions among the differentially expressed proteins, a protein–protein interaction (PPI) network was constructed using the STRING database (Figure 4C). The PPI network was divided into three major clusters. Cluster 1, the largest cluster comprising 43 proteins, was mainly associated with the complement and coagulation cascades. Cluster 2 consisted of 21 proteins and was associated with the complement and coagulation cascades and cholesterol metabolism. Cluster 3 consisted of HSP90AB1 and HSPB1, which formed a distinct interaction module related to the cellular stress response. These results suggest that chronic stress induces coordinated alterations in serum proteins involved in complement activation, coagulation, lipid metabolism, and stress responses. Functional classification analysis showed that the differentially expressed proteins were associated with several biological processes, including angiogenesis, apoptotic process, cell death, cell differentiation, cell migration, extracellular matrix organization, immune response, inflammatory response, neurogenesis, MAPK signaling, and secretion (Figure 4D). Notably, changes in proteins related to immune response, inflammatory response, and extracellular matrix organization, together with the PPI network results, suggest that chronic stress may modulate serum protein profiles associated with immune–inflammatory regulation, tissue remodeling, coagulation, lipid metabolism, and cellular stress responses.

### 3.3. Identification of Candidate Stress-Specific Biomarkers Based on Serum Proteomic Analysis

**Figure 5:**
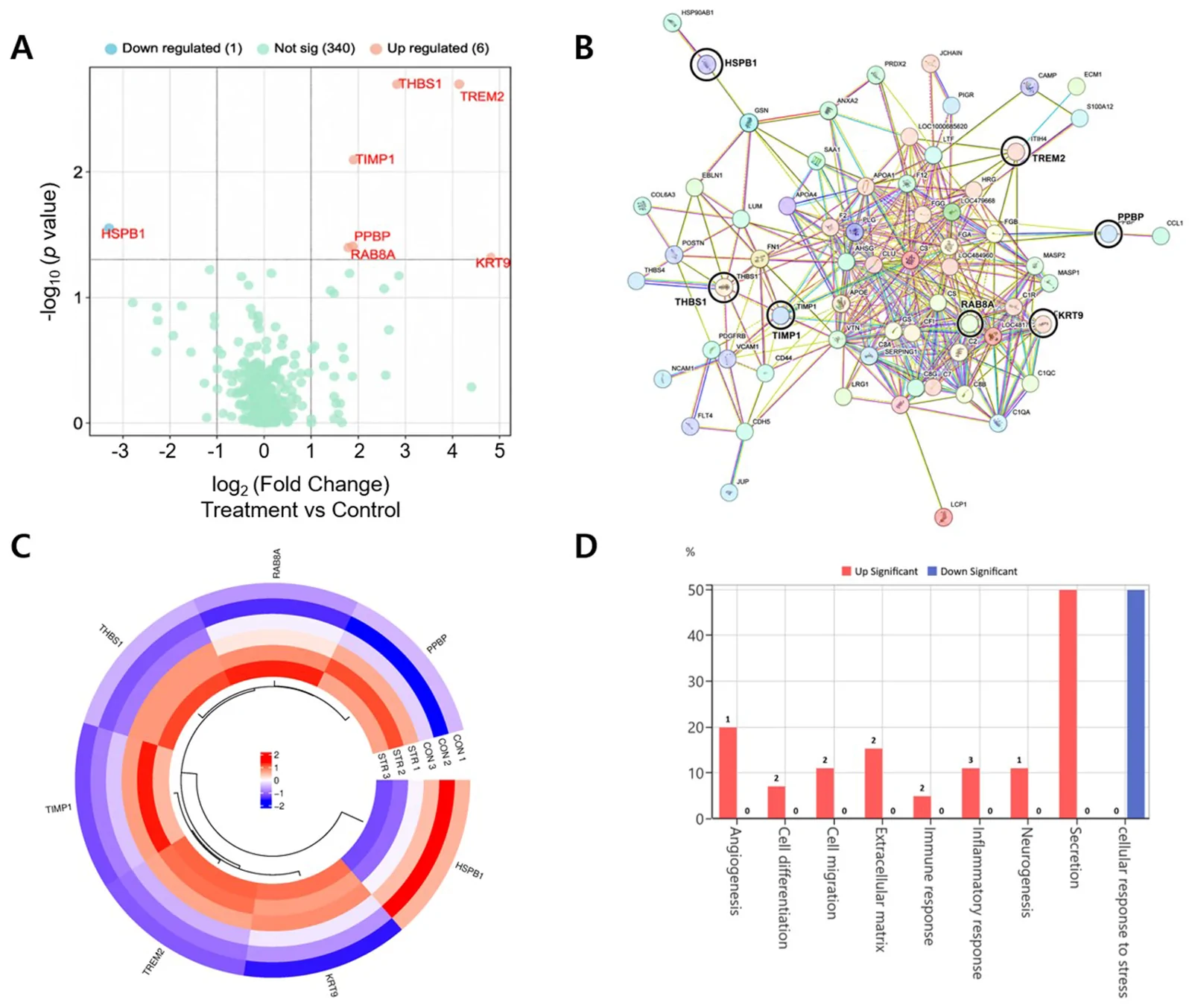
Identification and functional characterization of candidate stress-associated serum biomarkers. (**A**) Volcano plot showing differentially expressed proteins at day 42 compared with day 0 of the 84-day chronic stress model. Proteins with |log_2_FC| > 1 and *p* < 0.05 were considered significantly altered; red and blue dots indicate upregulated and downregulated proteins, respectively. The solid lines indicate the fold-change and statistical significance thresholds. (**B**) Protein–protein interaction network of the selected candidate proteins. (**C**) Circular heatmap showing relative abundance changes in the selected proteins between day 0 and day 42. (**D**) Functional classification of the selected candidate proteins.

To identify candidate stress-associated serum biomarkers, differentially expressed proteins were screened based on fold change and statistical significance. Proteins with ∣log2FC∣ > 1 and *p* < 0.05 were considered significantly altered. Among the selected proteins, seven proteins—thrombospondin 1 (THBS1), triggering receptor expressed on myeloid cells 2 (TREM2), tissue inhibitor of metalloproteinases 1 (TIMP1), pro-platelet basic protein (PPBP), Ras-related protein Rab-8A (RAB8A), and keratin 9 (KRT9)—were significantly upregulated, whereas heat shock protein family B member 1 (HSPB1) was significantly downregulated at day 42 compared with day 0 (Figure 5A). KRT9 showed the largest fold change, while THBS1 and TREM2 also showed marked increases. PPI analysis showed that most of the selected proteins were connected directly or indirectly within the interaction network (Figure 5B). THBS1, TIMP1, and RAB8A were located within the main network, whereas TREM2, PPBP, and KRT9 were positioned toward the outer regions. HSPB1 formed a separate interaction module with HSP90AB1. The circular heatmap showed distinct abundance patterns of the seven proteins between day 0 and day 42 of the 84-day chronic stress model (Figure 5C). THBS1, TREM2, TIMP1, PPBP, RAB8A, and KRT9 showed increased abundance at day 42 of the 84-day chronic stress model, whereas HSPB1 showed decreased abundance compared with day 0, consistent with the volcano plot results (Figure 5A). Functional classification analysis indicated that the selected proteins were associated with angiogenesis, cell differentiation, cell migration, extracellular matrix organization, immune response, inflammatory response, neurogenesis, secretion, and cellular response to stress (Figure 5D). Secretion showed the highest proportion of upregulated proteins, whereas cellular response to stress was represented by the downregulated protein HSPB1. Based on these results, TIMP1, TREM2, RAB8A, KRT9, PPBP, THBS1, and HSPB1 were selected as candidate stress-associated serum biomarkers in beagle dogs.

### 3.4. Effects of CZE on CBC Parameters in Chronically Stressed Beagle Dogs

**Figure 6:**
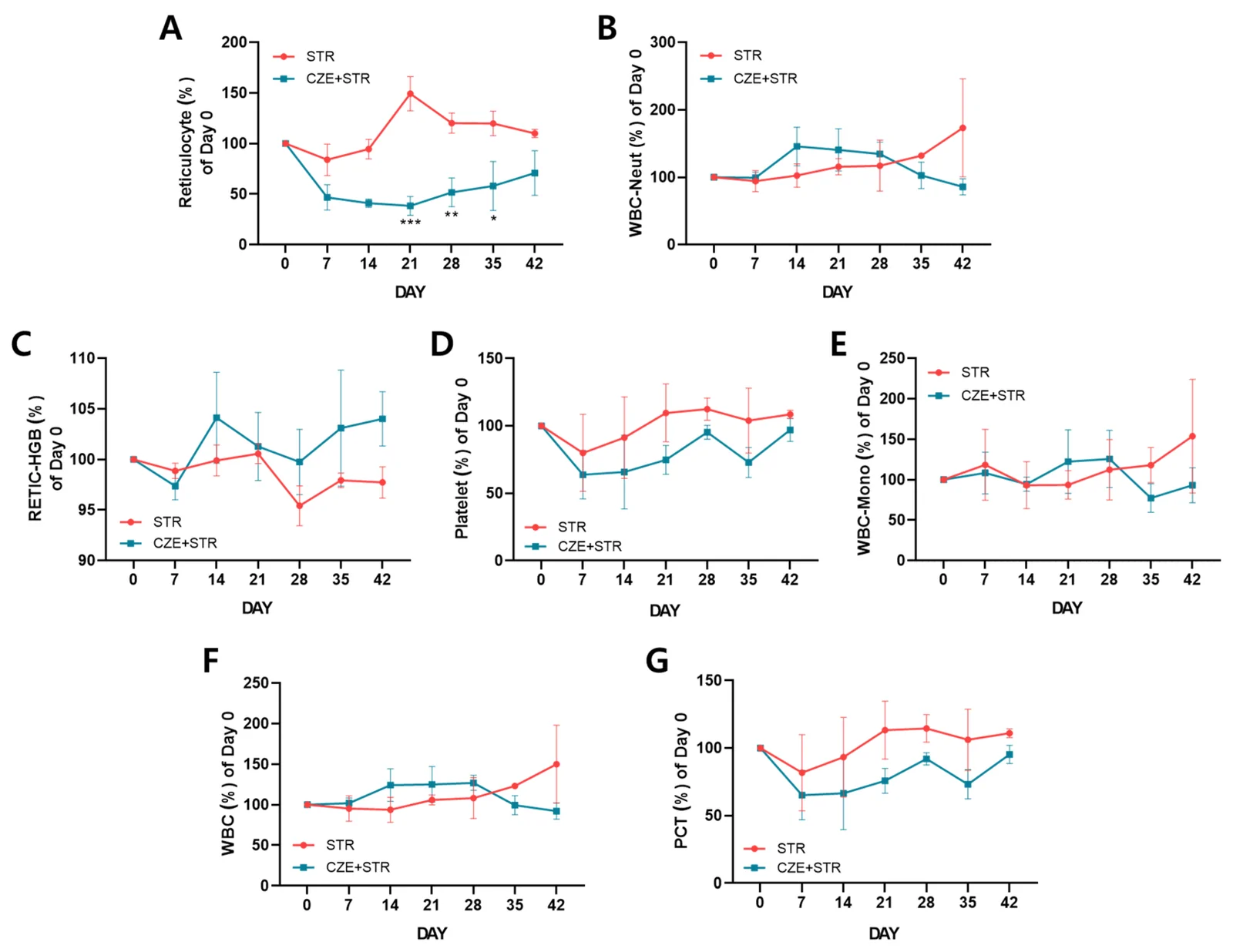
Effects of CZE on CBC parameters in chronically stressed beagle dogs. Beagle dogs were subjected to chronic stress for 42 days with or without repeated oral administration of CZE. Blood samples were collected at the indicated time points, and CBC analysis was performed to evaluate hematological changes. The analyzed parameters included (**A**) reticulocyte, (**B**) WBC-neutrophil, (**C**) RETIC-HGB, (**D**) platelet, (**E**) WBC-monocyte, (**F**) total WBC, and (**G**) plateletcrit (PCT). Data are presented as the mean ± SEM (*n* = 3 per group). Red and blue lines indicate the stress group and the CZE-treated group, respectively. Data were analyzed by two-way repeated-measures ANOVA with Dunnett’s test for comparisons with day 0 within the STR group and Sidak’s test for STR vs. CZE + STR comparisons at each time point. * *p* < 0.05, ** *p* < 0.01, *** *p* < 0.001 vs. STR at the corresponding time point.

To evaluate the hematological modulatory effects of CZE under chronic stress conditions, CBC analysis was performed using blood samples collected during the 42-day stress induction period with repeated oral administration of the extract. The analyzed CBC parameters included reticulocyte, white blood cell (WBC)-neutrophil, Reticulocyte Hemoglobin (RETIC-HGB), platelet, WBC-monocyte, and total WBC, plateletcrit (PCT). Reticulocyte levels tended to increase over time in the stress group, with relatively higher levels maintained during the mid-to-late phase of the experimental period (Figure 6A). In contrast, the CZE-treated group showed an attenuated increase in reticulocyte levels, maintaining relatively lower levels compared with the stress group (Figure 6A; Day 21, *p* < 0.001; Day 28, *p* = 0.07; Day 35, *p* = 0.017). WBC-neutrophil (Figure 6B; Day42, *p* = 0.246) and WBC-monocyte (Figure 6E; Day42, *p* = 0.690) also showed increasing or fluctuating patterns in the stress group, whereas these changes were relatively attenuated or stabilized in the CZE-treated group (Figure 6B,E). RETIC-HGB tended to remain at a lower level or decrease in the stress group, whereas the CZE-treated group maintained relatively higher levels (Figure 6C; Day 42, *p* = 0.516). In addition, platelet (Figure 6D; Day 42, *p* = 0.998) and PCT (Figure 6G; Day 42, *p* = 0.994) showed increasing trends during the later phase of stress induction in the stress group; however, these increases were relatively reduced or maintained at more stable levels following CZE administration (Figure 6D,G). Total WBC also tended to increase during the later phase in the stress group, while the CZE-treated group showed relatively lower and more stable levels (Figure 6F; Day 42, *p* = 0.215). Collectively, the CBC results suggest that chronic stress may induce alterations in hematopoietic, leukocyte-related, and platelet-related parameters in beagle dogs. Oral administration of CZE may contribute to the attenuation or stabilization of these stress-induced hematological changes.

### 3.5. Effects of CZE on Serum Biochemical Parameters in Chronically Stressed Beagle Dogs

**Figure 7:**
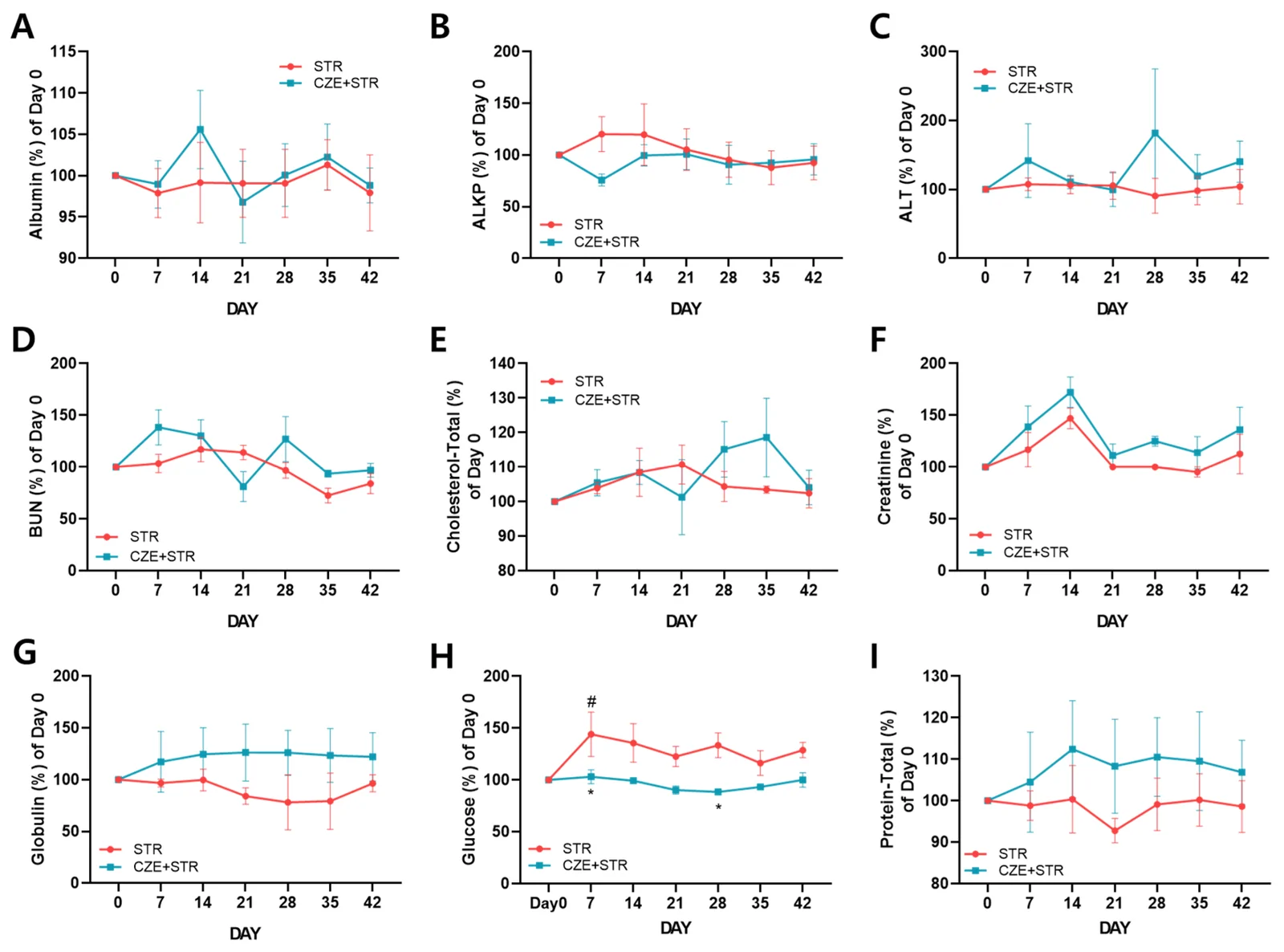
Effects of CZE on serum biochemical parameters in chronically stressed beagle dogs. Beagle dogs were subjected to chronic stress for 42 days with or without repeated oral administration of CZE. Serum samples were collected at the indicated time points, and serum chemistry analysis was performed to evaluate biochemical alterations. The analyzed parameters included (**A**) albumin, (**B**) alkaline phosphatase (ALKP), (**C**) alanine aminotransferase (ALT), (**D**) blood urea nitrogen (BUN), (**E**) total cholesterol, (**F**) creatinine, (**G**) globulin, (**H**) glucose, and (**I**) total protein. Data are presented as the mean ± SEM (*n* = 3 per group). Red and blue lines indicate the stress group and the CZE-treated group, respectively. Data were analyzed by two-way repeated-measures ANOVA with Dunnett’s test for comparisons with day 0 within the STR group and Sidak’s test for STR vs. CZE + STR comparisons at each time point. # *p* < 0.05 vs. STR day 0; * *p* < 0.05 vs. STR at the corresponding time point.

To evaluate the effects of CZE on stress-induced biochemical alterations, serum chemistry analysis was performed during the 42-day chronic stress induction period. The analyzed parameters included albumin, alkaline phosphatase (ALKP), alanine aminotransferase (ALT), blood urea nitrogen (BUN), total cholesterol, creatinine, globulin, glucose, and total protein. Albumin levels remained relatively stable in both groups, although transient fluctuations were observed during the experimental period (Figure 7A; Day 42, *p* = 0.997 vs. STR day0, *p* > 0.999 vs. STR). ALKP showed a mild increase in the stress group during the early-to-mid phase (Figure 7B; Day 7, *p* = 0.862; Day 14, *p* = 0.875; Day 42, *p* = 0.998), whereas the CZE-treated group maintained relatively stable levels (Figure 7B; Day 7, *p* = 0.31; Day42, *p* > 0.999). ALT (Figure 7C; Day 42, *p* = 0.986), BUN (Figure 7; Day 42, *p* = 0.979), and creatinine (Figure 7; Day 42, *p* = 0.785) showed time-dependent fluctuations in both groups without a consistent marked difference between the stress and CZE-treated groups. Total cholesterol tended to decrease in the stress group during the later phase of the experiment (Figure 7E; Day 42, *p* > 0.999), while the CZE-treated group showed relatively stable levels (Figure 7E; Day 42, *p* = 0.992). In addition, globulin and total protein tended to remain lower in the stress group (Figure 7G; Day 42, *p* > 0.999 and 7I; Day 42, *p* > 0.999), whereas these parameters were relatively maintained in the CZE-treated group (Figure 7G; Day 42, *p* = 0.963 and 7I; Day 42, *p* = 0.989). Glucose levels showed an increasing tendency in the stress group after stress induction (Figure 7H; Day 7, *p* = 0.018; Day 42, *p* = 0.191), while the CZE-treated group exhibited relatively attenuated changes (Figure 7H; Day 7, *p* = 0.044; Day 28, *p* = 0.021; Day 42, *p* = 0.289). Collectively, these results suggest that chronic stress induced alterations in serum biochemical profiles, particularly in parameters associated with serum protein balance, lipid metabolism, and glucose regulation. Oral administration of CZE may contribute to the stabilization of stress-induced serum biochemical changes in beagle dogs.

### 3.6. Effects of CZE on Serum Cortisol and Corticosterone Levels in Chronically Stressed Beagle Dogs

**Figure 8:**
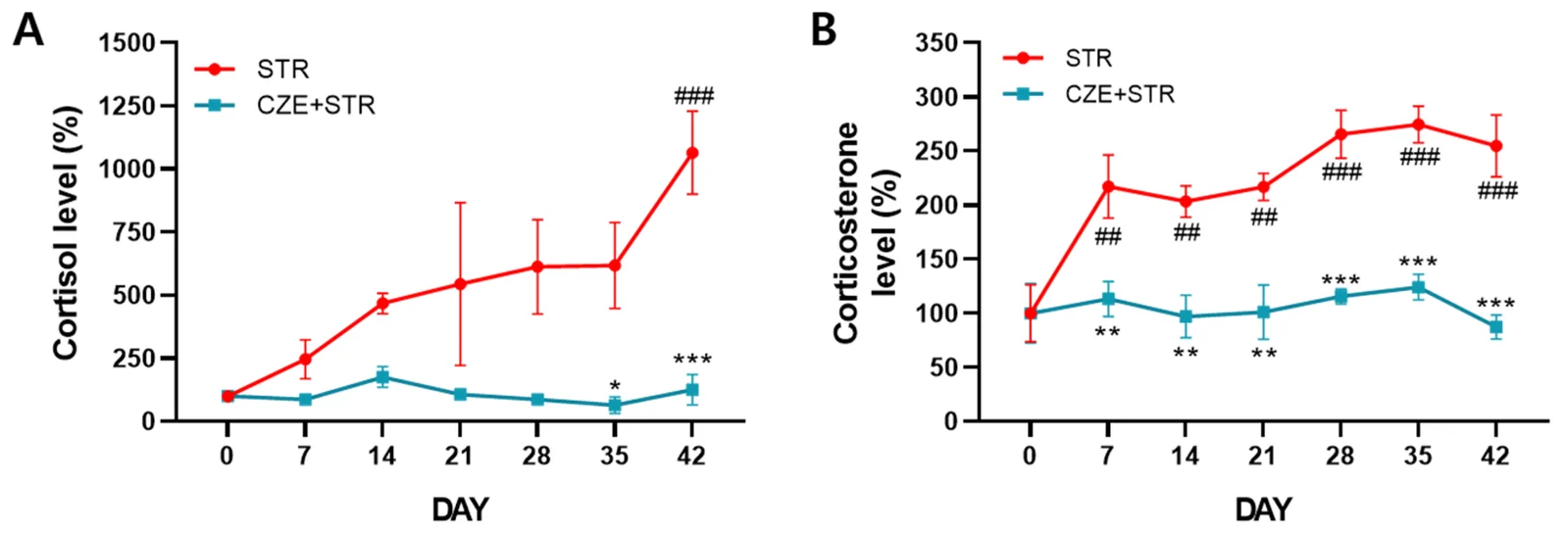
Effects of CZE on serum cortisol and corticosterone levels in chronically stressed beagle dogs. Beagle dogs were subjected to chronic stress for 42 days with or without repeated oral administration of CZE. Serum samples were collected at indicated time points, and cortisol and corticosterone concentrations were measured using canine-specific ELISA kits. (**A**) Serum cortisol levels. (**B**) Serum corticosterone levels. Data are presented as the mean ± SEM (*n* = 3 per group). Red and blue lines indicate the stress group and the CZE-treated group, respectively. Data were analyzed by two-way repeated-measures ANOVA with Dunnett’s test for comparisons with day 0 within the STR group and Sidak’s test for STR vs. CZE + STR comparisons at each time point. ## *p* < 0.01, ### *p* < 0.001 vs. STR day 0; * *p* < 0.05, ** *p* < 0.01, and *** *p* < 0.001 vs. STR at the corresponding time point.

To evaluate the anti-stress effects of CZE under chronic stress conditions, serum cortisol and corticosterone concentrations were measured using canine-specific ELISA kits during the 42-day stress induction period with repeated oral administration of the extract. Serum cortisol levels showed a gradual time-dependent increase in the stress group over the experimental period, with the highest levels observed during the later phase of stress induction (Figure 8A; Day 42, *p* < 0.001). In contrast, the CZE-treated group showed a marked attenuation of cortisol elevation, maintaining relatively lower cortisol levels compared with the stress group throughout the experimental period (Figure 8A; Day 35, *p* = 0.024; Day 42, *p* < 0.001). These results suggest that oral administration of CZE may suppress or stabilize the stress-induced increase in serum cortisol levels. Similarly, serum corticosterone levels increased rapidly after stress induction in the stress group and remained elevated during the experimental period (Figure 8B; Day 7, *p* = 0.002; Day 14, *p* = 0.007; Day 21, *p* = 0.002; Day 28–42, *p* < 0.001). However, the CZE-treated group exhibited relatively lower corticosterone levels compared with the stress group (Figure 8B; Day 7, *p* = 0.009; Day 14, *p* = 0.007; Day 21, *p* = 0.003; Day 28–42, *p* < 0.001), indicating that CZE administration attenuated the sustained increase in corticosterone induced by chronic stress. Collectively, ELISA analysis demonstrated that chronic stress increased serum glucocorticoid levels, including cortisol and corticosterone, in beagle dogs. Repeated oral administration of CZE reduced or stabilized these stress-induced glucocorticoid elevations, suggesting that the extract may exert anti-stress effects by modulating HPA axis-associated endocrine responses under chronic stress conditions.

## 4. Discussion

In the present study, we established a movement restriction-based chronic stress model in beagle dogs and comprehensively characterized stress-associated alterations at the endocrine, hematological, biochemical, and serum proteomic levels. Serum cortisol and corticosterone concentrations progressively increased during the stress induction period [2] and remained elevated through day 42 (Figure 3A,B).

Cortisol and corticosterone are major glucocorticoids released following activation of the HPA axis and represent important endocrine indicators of the physiological stress response [29,30]. Although transient increases in glucocorticoid concentrations may occur in response to acute handling or environmental stimuli, persistent elevation during repeated stress exposure is more consistent with sustained activation of the HPA axis [7,29,30]. In addition to serving as stress indicators, these hormones regulate energy metabolism, immune activity, inflammatory responses, and overall physiological homeostasis [29,31]. Therefore, prolonged changes in circulating cortisol and corticosterone may affect physiological functions beyond the endocrine system.

The concurrent and sustained increases in cortisol and corticosterone supported the validity of the established chronic stress model. Based on these endocrine profiles, day 42 was selected as the representative time point for serum proteomic analysis because both glucocorticoids remained elevated, indicating that a chronic stress state had been established and that downstream systemic and molecular alterations could be evaluated. In addition to glucocorticoid elevation, chronic stress was accompanied by alterations in multiple peripheral blood and serum biochemical parameters [21]. During model establishment, reticulocyte and albumin levels showed decreasing trends, whereas ALT and total cholesterol tended to increase (Figure S1). Although these parameters are not individually specific to stress, their coordinated changes suggest that the stress response extended beyond the endocrine system and involved multiple physiological processes [32].

A major finding of this study was the extensive remodeling of the serum proteome under chronic stress conditions. Among the 380 identified serum proteins, 348 quantified proteins were analyzed, and the differentially expressed proteins were functionally associated with immune response, inflammatory response, extracellular matrix organization, angiogenesis, neurogenesis, cell migration, and secretion (Figure 4A,B,D and Figure S2A). PPI network analysis further demonstrated that the differentially expressed proteins formed interconnected modules primarily associated with complement and coagulation cascades, cholesterol metabolism, and cellular stress responses (Figure 4C). These findings indicate that chronic stress affects circulating protein networks associated with immune–inflammatory regulation, coagulation, lipid metabolism, cellular stress adaptation, and tissue remodeling, supporting the utility of serum proteomics for identifying molecular signatures of prolonged stress exposure [33,34].

Based on differential expression, statistical significance, PPI network connectivity, and functional relevance, TIMP1, TREM2, RAB8A, KRT9, PPBP, THBS1, and HSPB1 were selected as candidate stress-associated serum biomarkers (Figure 5 and Figure S3B). The functional characteristics of these proteins indicate that they may represent several interconnected responses to prolonged stress, including immune–inflammatory regulation, extracellular matrix remodeling, platelet-associated signaling, intracellular trafficking, and cellular protein homeostasis (Figure 5D and Figure S3A). TIMP1 and THBS1 are closely linked to extracellular matrix remodeling, cell migration, and tissue repair [35,36], suggesting that their altered abundance under chronic stress may be associated with changes in tissue remodeling and extracellular matrix-related signaling. Prolonged glucocorticoid elevation can influence inflammatory regulation, vascular function, and tissue homeostasis [37,38]. Accordingly, the increased serum abundance of TIMP1 (Figure 5A,C) may reflect compensatory regulation of extracellular matrix turnover in response to chronic stress-associated tissue and inflammatory changes. This interpretation is consistent with the enrichment of extracellular matrix organization, cell migration, and inflammatory response categories (Figure 5D and Figure S3A). THBS1 is a matricellular protein released from platelets and involved in cell adhesion, angiogenesis, inflammatory signaling, and extracellular matrix remodeling [36]. In the STR group, platelet and plateletcrit levels generally remained higher than those in the CZE + STR group during the later stages of stress exposure (Figure 6D,G). Therefore, the increased abundance of THBS1 may reflect platelet-associated signaling and vascular or extracellular matrix remodeling activated during prolonged stress (Figure 6D,G). The enrichment of angiogenesis, extracellular matrix organization, and coagulation-related pathways further supports this interpretation (Figure 4B–D, Figure 5D and Figure S3A).

TREM2 participates in macrophage-associated inflammatory regulation, phagocytosis, and the clearance of damaged or stressed cells [39]. Under conditions in which steady-state erythropoiesis is insufficient or disrupted, stress erythropoiesis can increase erythroid output and the release of immature erythroid cells into the circulation [40,41]. In the present study, reticulocyte levels increased in the STR group, whereas this increase was attenuated in the CZE + STR group (Figure 6A). Given the role of TREM2 in macrophage-mediated phagocytosis, the increased serum abundance of TREM2 (Figure 5A,C) may reflect enhanced myeloid cell activity associated with the clearance or turnover of stress-affected erythroid cells. However, because the direct interaction between TREM2-expressing macrophages and circulating reticulocytes was not examined, this interpretation remains hypothetical. PPBP, also known as CXCL7, is a platelet-derived chemokine that promotes neutrophil recruitment and contributes to communication between platelets and immune cells [42,43]. In the present study, platelet and plateletcrit levels were generally elevated in the STR group during prolonged stress exposure (Figure 6D,G), whereas WBC-neutrophil, WBC-monocyte, and total WBC levels showed increasing or fluctuating patterns during the later stages of the experiment (Figure 6B,E,F). The increased serum abundance of PPBP may therefore reflect enhanced platelet–leukocyte communication during chronic stress.

RAB8A is involved in intracellular vesicle trafficking and secretory pathways [44,45]. Chronic activation of the HPA axis can alter glucose and lipid metabolism during prolonged stress [46]. In the present study, serum glucose increased markedly in the STR group, while total cholesterol showed temporal changes (Figure 7E,H). Together with the enrichment of secretion- and cholesterol metabolism-related pathways (Figure 4C,D and Figure S3A), the increased abundance of RAB8A may reflect altered cellular transport, secretion, and metabolic adaptation during chronic stress. However, a direct relationship between RAB8A and the observed metabolic changes was not established. KRT9 is a structural keratin involved in maintaining the mechanical stability of differentiated epithelial tissues [47]. Its increased serum abundance may reflect epithelial turnover or the release of tissue-derived structural proteins. Nevertheless, because KRT9 is primarily associated with specialized epidermal tissues [47] and no skin or epithelial tissue analysis was conducted, its cellular origin and direct relevance to chronic stress remain uncertain.

HSPB1, also known as HSP27, is a small heat shock protein involved in molecular chaperone activity, proteostasis, cytoskeletal stabilization, and cellular protection against oxidative and proteotoxic stress [48,49]. Unlike the other candidate proteins, HSPB1 showed decreased abundance in the chronic stress group, which may indicate altered cellular stress adaptation and protein homeostasis during prolonged stress exposure. This decrease occurred in the presence of progressively elevated cortisol and corticosterone concentrations (Figure 8A,B) and sustained alterations in glucose and circulating protein profiles (Figure 7A,G–I), indicating that prolonged stress was accompanied by both endocrine and metabolic disturbances. Although HSPB1 is commonly induced in response to acute cellular stress, including heat shock and oxidative stress, its decreased abundance after prolonged stress exposure may reflect altered or insufficient maintenance of cytoprotective and protein-homeostasis mechanisms during chronic stress [49,50]. The PPI network showed that most of the selected proteins were directly or indirectly connected within the major interaction network, whereas HSPB1 formed a relatively distinct interaction module with HSP90AB1 (Figure 5B). This relatively distinct interaction pattern further suggests that HSPB1 may represent a cellular stress-adaptation response that differs from the immune–inflammatory, platelet-associated, and extracellular matrix-related responses represented by the other candidate proteins.

Together with the enrichment of immune–inflammatory and extracellular matrix-related functional categories (Figure 5D and Figure S3A), these expression patterns indicate that prolonged stress was associated with coordinated changes in tissue remodeling, inflammatory regulation, platelet-associated signaling, intracellular trafficking, metabolic adaptation, and cellular stress responses [51]. These protein changes occurred alongside sustained glucocorticoid elevation and alterations in hematological and serum biochemical profiles, suggesting that the identified proteins collectively reflect systemic physiological responses to chronic stress rather than changes in a single pathway. Nevertheless, because serum proteomics reflects circulating protein abundance rather than tissue-specific expression, the biological sources and mechanistic roles of these proteins remain to be clarified [52]. Moreover, the associations between the candidate proteins and the observed hematological and biochemical changes were not directly evaluated through correlation or mechanistic analyses. Accordingly, the proteins identified in this study should be considered candidate biomarkers rather than definitive indicators or mediators of chronic stress. In addition to characterizing stress-associated serum protein changes, the present study evaluated the potential anti-stress effects of CZE under prolonged stress conditions. Repeated oral administration of CZE tended to stabilize several hematological parameters, including platelet-related indices and leukocyte populations (Figure 6). Additional erythrocyte-, platelet-, and leukocyte-related parameters also showed relatively limited or stabilized variations following CZE administration (Figure S4). These findings suggest that CZE administration may reduce stress-associated fluctuations in peripheral blood parameters during prolonged stress exposure.

CZE administration was also associated with changes in serum biochemical parameters related to serum protein balance, lipid metabolism, glucose regulation, and organ-associated biochemical responses (Figure 7). Additional metabolic and organ-associated biochemical parameters were monitored throughout the experimental period (Figure S5). Although some parameters showed temporal fluctuations, the combined hematological and biochemical findings suggest that CZE may contribute to the maintenance of physiological homeostasis during chronic stress exposure. Repeated oral administration of CZE also tended to attenuate stress-associated increases in serum cortisol and corticosterone concentrations (Figure 8A,B). Because these glucocorticoids were the principal endocrine indicators used to confirm establishment of the chronic stress model, their attenuation following CZE administration further supports the potential anti-stress activity of CZE. However, because these changes were observed primarily as trends, further studies with larger sample sizes and additional HPA-axis-related measurements are required to determine the magnitude and reproducibility of this effect. A limitation of the present study is the heterogeneity in the age and sex of the dogs, which may have contributed to inter-individual variability and potentially influenced the physiological and biochemical responses observed in this study. Future studies using larger and more homogeneous populations or stratified analyses according to age and sex are warranted to further validate these findings.

Taken together, chronic stress in beagle dogs was associated with sustained glucocorticoid elevation, alterations in hematological and serum biochemical profiles, and broad remodeling of the serum proteome. The identified proteins represent candidates for future biomarker validation, although their tissue origins and mechanistic roles require further investigation. Overall, the stabilization of hematological and biochemical profiles and the attenuation of glucocorticoid responses suggest that CZE may have potential as a functional material for alleviating physiological alterations associated with chronic stress.

## 5. Conclusions

Collectively, this study demonstrates that chronic stress in beagle dogs is associated with sustained glucocorticoid elevation consistent with activation of the HPA axis, alterations in hematological and serum biochemical parameters, and broad changes in the serum proteomic profile. CZE administration tended to attenuate stress-associated changes in glucocorticoid levels and partially stabilized hematological and biochemical parameters under prolonged stress conditions, supporting its potential as a functional anti-stress material. In addition, the stress-associated serum protein candidates identified through proteomic analysis may provide a useful basis for the development of chronic stress biomarkers and the evaluation of natural product-based anti-stress strategies.

## Data Availability

The original contributions presented in this study are included in the article/Supplementary Materials. Further inquiries can be directed to the corresponding author.

## Supplementary Materials

The following supporting information can be downloaded at https://www.mdpi.com/article/10.3390/ani16172679/s1. Table S1: Formulation composition of CZE tablets according to the blending process; Table S2: Characteristics of beagle dogs used in the chronic stress model establishment study; Table S3: Characteristics of beagle dogs used in the CZE efficacy study; Table S4: List of blood test parameters; Table S5: LC-MS/MS acquisition conditions; Table S6: Database search parameters for protein identification; Figure S1: Changes in CBC and serum biochemical parameters following chronic stress induction; Figure S2: Functional classification and fold-change heatmap of differentially abundant serum proteins under chronic stress conditions; Figure S3: Functional classification and abundance distribution of candidate stress-associated serum proteins; Figure S4: Changes in hematological parameters in stress-induced beagle dogs; Figure S5: Changes in serum biochemical parameters in stress-induced beagle dogs.

## Abbreviations

The following abbreviations are used in this manuscript:

STR	Stress
HPA-axis	Hypothalamic–pituitary–adrenal axis
CZ	*Chrysanthemum zawadskii* var. *latilobum* (Maxim.) Kitam.
CZE	*Chrysanthemum zawadskii* var. *latilobum* (Maxim.) Kitam. extract
THBS1	Thrombospondin 1
TREM2	Triggering receptor expressed on myeloid cells 2
TIMP1	Tissue inhibitor of metalloproteinases 1
HSPB1	Heat shock protein beta 1
RAB8A	Ras-related protein Rab-8A
KRT9	Keratin 9
PPBP	Pro-platelet basic protein
EDTA	Ethylenediaminetetraacetic acid
LC-MS/MS	Liquid chromatography–tandem mass spectrometry
MS/MS	Tandem mass spectrometry
ELISA	Enzyme-Linked Immunosorbent Assay
CBC	Complete blood count
SST	Serum-separating tubes
DR	Dissociation reagent
PCT	Plateletcrit
ALKP	Alkaline phosphatase
ALT	Alanine aminotransferase
BUN	Blood urea nitrogen
WBC	White blood cell
BSA	Bovine serum albumin
BCA	Bicinchoninic acid
RETIC-HGB	Reticulocyte hemoglobin
