# Supplementary material for: Physiological and Proteomic Responses to Chronic Stress and the Effects of Chrysanthemum zawadskii var. latilobum (Maxim.) Kitam. Extract in Beagle Dogs
Source: Animals (Basel). 2026 Aug 26;16(17):2679. doi: 10.3390/ani16172679 (PMC13564297; doi:10.3390/ani16172679)
Supplement: Supplementary file 1 [file animals-16-02679-s001.zip › animals-4487497-supplementary.pdf]

Supplementary Materials

Supplementary Table S1. Formulation composition of CZE tablets according to the blending process.

Process	Materials	Amount (mg/tablet)	Proportion (%)
First blending	CZE	200.0	43.5
	SiO_2	15.0	3.3
	Avicel PH102	200.0	43.5
	Copovidone	20.0	4.3
	Cros CMC Na	20.0	4.3
Final blending	S-Mg	5.0	1.1
Total		460.0	100

Supplementary Table S2. Characteristics of beagle dogs used in the chronic stress model establishment study.

Breed	Number of dogs	Age	Sex
Beagle	4	9 years	Male
	2	3 years	Male
	4	9 months	Female

Supplementary Table S3. Characteristics of beagle dogs used in the CZE efficacy study.

Breed	Number of dogs	Age	Sex
Beagle	2	7 years	Male
	2	4 years	Male
	1	1 year	Male
	1	1 year	Female

Supplementary Table S4. List of blood test parameters.

List	
**CBC**	**Serum Chemistry**
Hematocrit (Hct)	Albumin
Hemoglobin (Hb)	ALKP
MCH	ALT
MCHC	Amylase
MCV	BUN
MPV	Calcium
PCT	Total Cholesterol
Platelet	Creatinine
RBC	Globulin
Reticulocyte (#)	Glucose
Reticulocyte (%)	Lipase
WBC	Phosphorus-Inorganic
WBC-Baso (#)	Protein-Total
WBC-Eos (#)	ALB/GLOB
WBC-Eos (%)	BUN/CREA
WBC-Lymph (#)	
WBC-Lymph (%)	
WBC-Mono (#)	
WBC-Mono (%)	
WBC-Neut (#)	
WBC-Neut (%)	
RDW	
PDW	
RETIC-HGB	

Supplementary Table S5. LC-MS/MS acquisition conditions.

Parameters	Conditions						
Trapping Column	Acclaim PepMapTM 100, C18, 5 µm, 100 Å, 100 µm × 2 cm						
Analytical Column	PepMapTM RSLC C18 2 µm, 100 Å, 75 µm × 50 cm						
Mobile Phase	A: 0.1% formic acid in 5% DMSO<br>B: 0.1% formic acid in 5% DMSO and 80% ACN						
Gradient	Time (min)	0	150	152	175	185	200
	Solvent B(%)	5	40	95	95	5	5
Column Flow Rate	400 nL/min						
Mass Range							

Supplementary Table S6. Database search parameters for protein identification.

Parameters	Variable modification
Database	UniProt *Canis lupus familiaris*
Software	Proteome Discoverer 3.0
Fixed modification	Carbamidomethylation of cysteine
Variable modification	Oxidation of methionine
	Carbamylation of protein N-terminus
	Acetylation of protein N-terminus

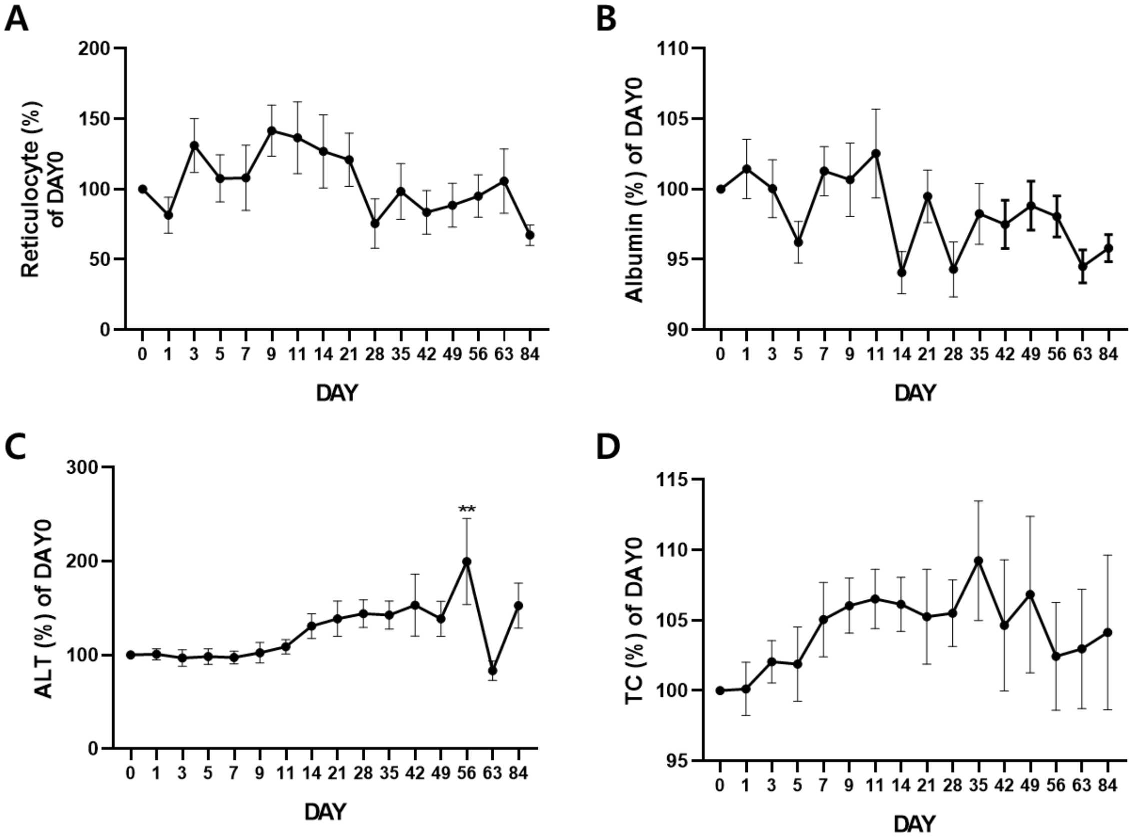

Supplementary Figure S1. Changes in CBC and serum biochemical parameters following chronic stress induction. Complete blood count (CBC) and serum chemistry analyses were performed to evaluate hematological and serum biochemical changes in beagle dogs subjected to chronic stress. (A) Changes in reticulocyte levels measured by CBC analysis. (B–D) Changes in albumin, ALT, and total cholesterol measured by serum chemistry analysis. Data are expressed as a percentage of the control group and presented as the mean ± SEM (n=10). Reticulocyte and albumin levels showed overall decreasing trends during the stress induction period, whereas ALT and total cholesterol tended to increase under chronic stress conditions. Data were analyzed by two-way repeated-measures ANOVA with Dunnett's test for comparisons with day 0 within the STR group. ***P* < 0.01 vs. STR day 0.

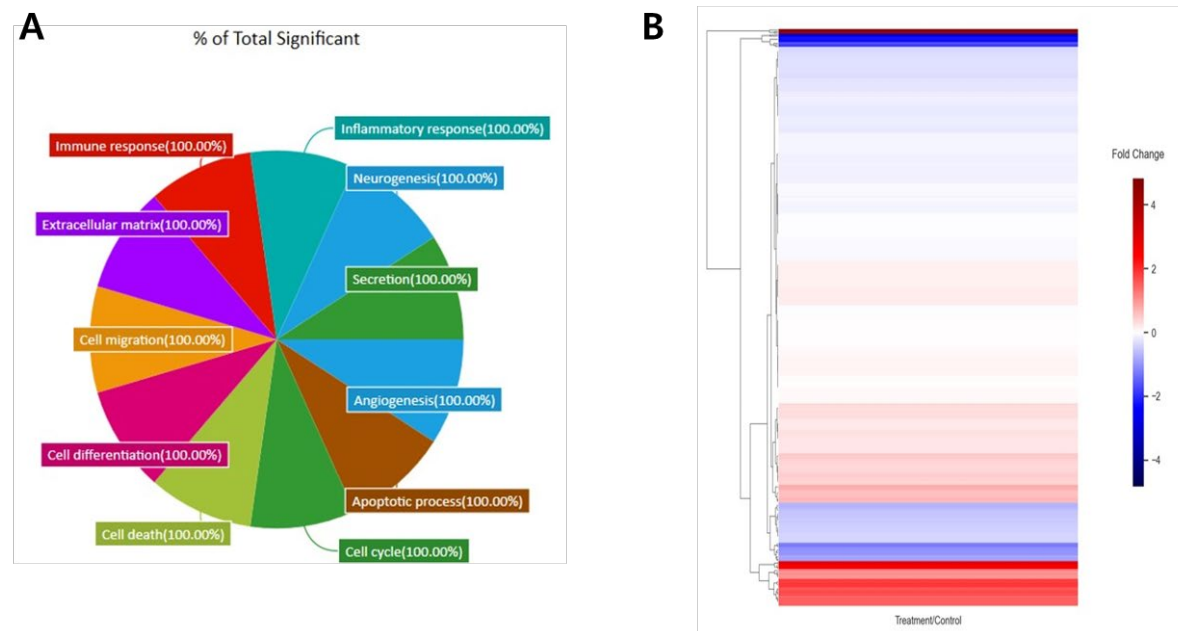

Supplementary Figure S2. Functional classification and fold-change heatmap of differentially abundant serum proteins under chronic stress conditions. Differentially expressed serum proteins identified by LC-MS/MS-based proteomic analysis of day 42 canine serum samples were further analyzed for functional classification and fold-change patterns. **(A)** Functional classification pie chart of differentially expressed proteins based on biological process categories. The classified categories include angiogenesis, apoptotic process, cell death, cell differentiation, cell migration, extracellular matrix, immune response, inflammatory response, neurogenesis, and secretion. **(B)** Fold-change heatmap showing relative protein abundance changes in the stress group compared with the control group. Red indicates relatively increased protein abundance, whereas blue indicates relatively decreased protein abundance.

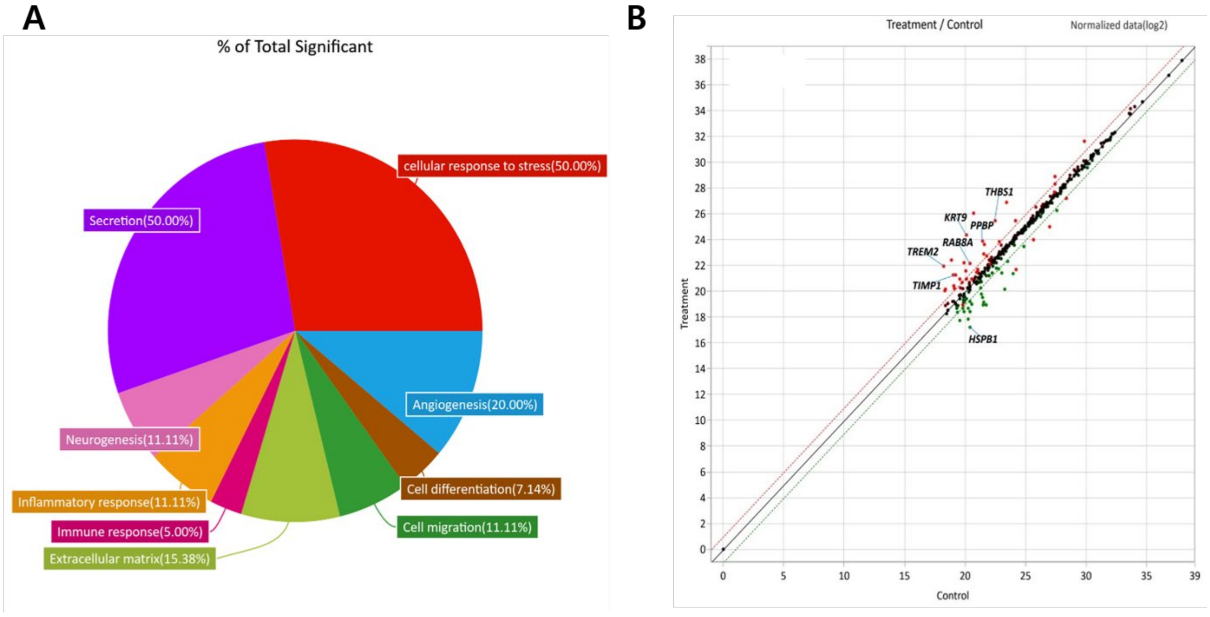

Supplementary Figure S3. Functional classification and abundance distribution of candidate stress-associated serum proteins. (A) Functional classification of selected candidate stress-associated proteins based on biological process categories. (B) Scatter plot showing normalized protein abundance between the control and stress groups. Each dot represents an individual protein, and labeled dots indicate selected candidate proteins. Proteins above the diagonal line indicate increased abundance in the stress group, whereas proteins below the diagonal line indicate decreased abundance.

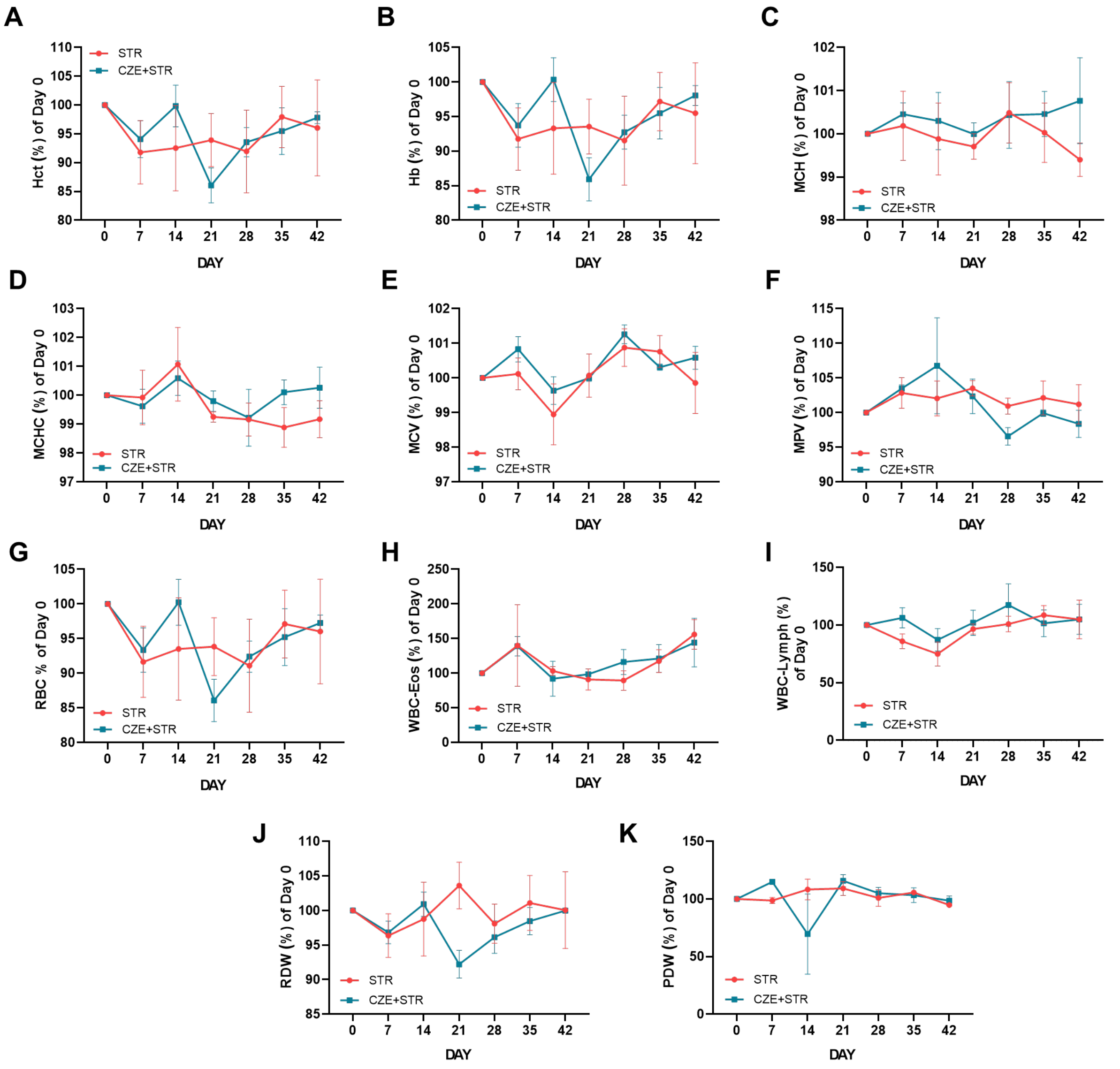

Supplementary Figure S4. Changes in hematological parameters in stress-induced beagle dogs. Hematological changes were evaluated by complete blood count analysis during the experimental period in the stress-only group (STR) and the CZE-treated stress group (CZE+STR). Each parameter is expressed as a percentage of the Day 0 value. (A) Hematocrit (Hct), (B) hemoglobin (Hb), (C) mean corpuscular hemoglobin (MCH), (D) mean corpuscular hemoglobin concentration (MCHC), (E) mean corpuscular volume (MCV), (F) mean platelet volume (MPV), (G) red blood cell count (RBC), (H) eosinophil count (WBC-Eos), (I) lymphocyte count (WBC-Lymph), (J) red cell distribution width (RDW), and (K) platelet distribution width (PDW). Data are presented as the mean ± SEM (n=3 per group). Red and blue lines indicate the stress group and the CZE-treated group, respectively. Data were analyzed by two-way repeated-measures ANOVA with Dunnett's test for comparisons with day 0 within the STR group and Sidak's test for STR vs. CZE+STR comparisons.

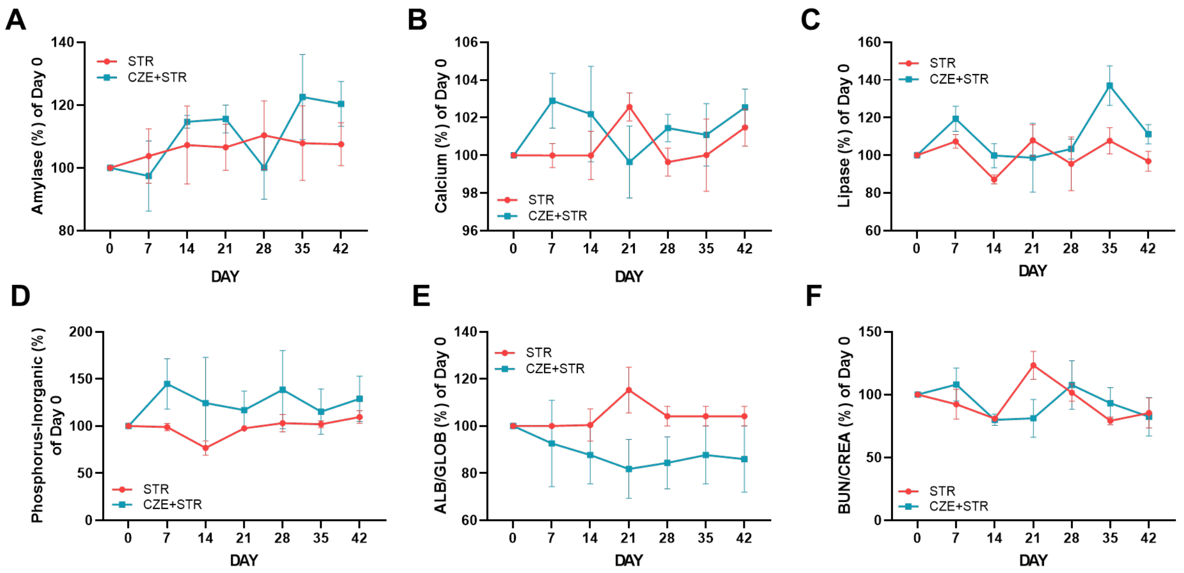

Supplementary Figure S5. Changes in serum biochemical parameters in stress-induced beagle dogs. Changes in serum biochemical parameters were evaluated during the experimental period in the stress-only group (STR) and the CZE-treated stress group (CZE+STR). Each parameter is expressed as a percentage of the Day 0 value. (A) Amylase, (B) calcium, (C) lipase, (D) inorganic phosphorus, (E) albumin/globulin ratio (ALB/GLOB), and (F) blood urea nitrogen/creatinine ratio (BUN/CREA). Data are presented as the mean ± SEM (n=3 per group). Red and blue lines indicate the stress group and the CZE-treated group, respectively. Data were analyzed by two-way repeated-measures ANOVA with Dunnett's test for comparisons with day 0 within the STR group and Sidak's test for STR vs. CZE+STR comparisons at each time point.
